# An Adaptive Damping Network Designed for Strapdown Fiber Optic Gyrocompass System for Ships

**DOI:** 10.3390/s17030494

**Published:** 2017-03-02

**Authors:** Jin Sun, Xiaosu Xu, Yiting Liu, Tao Zhang, Yao Li, Jinwu Tong

**Affiliations:** Key Laboratory of Micro-Inertial Instrument and Advanced Navigation Technology, Ministry of Education, School of Instrument Science and Engineering, Southeast University, Nanjing 210096, China; sunjin8607986@126.com (J.S.); 230119509@seu.edu.cn (Y.L.); 101011356@seu.edu.cn (T.Z.); liyao@seu.edu.cn (Y.L.); 230139522@seu.edu.cn (J.T.)

**Keywords:** strapdown fiber optic gyrocompass (strapdown FOGC), adaptive damping network, overshoot error, external horizontal damping, dynamic performance

## Abstract

The strapdown fiber optic gyrocompass (strapdown FOGC) system for ships primarily works on external horizontal damping and undamping statuses. When there are large sea condition changes, the system will switch frequently between the external horizontal damping status and the undamping status. This means that the system is always in an adjustment status and influences the dynamic accuracy of the system. Aiming at the limitations of the conventional damping method, a new design idea is proposed, where the adaptive control method is used to design the horizontal damping network of the strapdown FOGC system. According to the size of acceleration, the parameters of the damping network are changed to make the system error caused by the ship’s maneuvering to a minimum. Furthermore, the jump in damping coefficient was transformed into gradual change to make a smooth system status switch. The adaptive damping network was applied for strapdown FOGC under the static and dynamic condition, and its performance was compared with the conventional damping, and undamping means. Experimental results showed that the adaptive damping network was effective in improving the dynamic performance of the strapdown FOGC.

## 1. Introduction

There are five kinds of working statuses: Undamping, internal horizontal damping, internal whole damping, external horizontal damping, and external whole damping in the strapdown fiber optic gyrocompass （FOGC）system [1]. For the strapdown FOGC system with long working time, it mainly works on external horizontal damping and undamping status. In order to adapt to different navigational statuses, the strapdown FOGC system needs to frequently switch between the external horizontal damping status and undamping status. Furthermore, the parameters of the horizontal damping network adopted in the practical application of the strapdown FOGC system for ships often are of fixed value, and it is therefore difficult to adapt to the damping needs of different accelerations. When a ship sails straight with a constant velocity, the system works on the external horizontal damping status; when the ship maneuvers, the system works on the undamping status through the status switch. In this case, when there are larger changes in sea conditions, the system will frequently switch between the external horizontal damping and undamping statuses, which means that the system is always in an adjustment status. Currently, there is a large overshoot error as the balance status of the system is destroyed, resulting in reduced system accuracy [2]. Therefore, optimizing the design of damping networks is of great theoretical and practical significance to improve the accuracy of navigation systems [3].

With regard to the design for the damping network of inertial navigation system (INS), the conventional design method is based on classical control theory, and the method of trying step by step is used; for example, a first order damping network is given in Reference [4]. In References [5,6], a two order horizontal damping network was discussed, and the parameters of the damping network were of a fixed value. In Reference [7], the velocity, position, and acceleration errors of two sets of INS feedbacks to the Schuler loop formed a damping network, but required two sets of INS. In Reference [8], they aimed at the problem of the Earth cycle oscillation component of latitude and azimuth error which can reduce the accuracy of INS, and a three order damping network was introduced and the azimuth damping network with many damping coefficients was designed through trial and error. A two-order damping network was designed in Reference [9], where a fuzzy controller was used to judge the motion state of the carrier at the current time and the data fusion strategy was used to control the switch between the undamping and internal damping status. In Reference [10], a horizontal damping discretization algorithm with a bilinear transformation was designed and was based on the three order compass alignment network, in combination with the selection principle of the horizontal damping network in strict accordance with the requirements of the dominant pole placement method. An optimal two-order horizontal damping network with various damping coefficients was given in References [11,12]. In Reference [13], a novel damped compensation algorithm was presented. According to control theory, the system added three different damp feedbacks in three loops, which made use of its own velocity information to perform compensation for errors. A technique of adaptive robust (AR) damping of oscillations in the gyro-accelerometer system was proposed in Reference [14]. In References [15,16], based on modern control theory, a new method used Kalman filtering and feedback calibration to damp the Schuler oscillation on INS. A kind of fuzzy internal damping algorithm was introduced in References [17,18,19], according to fuzzy rules, where three-axis accelerometer information was used to determine the motion state of the system in real time, before accurately controlling the use of the internal damping algorithm. An adaptive network based on fuzzy inference system (ANFIS) for strapdown FOGC was proposed in Reference [20]. ANFIS was utilized to adjust the damping ratio automatically in terms to the ship maneuver conditions. A geometric method, based on a tetrahedral configuration to obtain a deterministic fix for position, is proposed in References [21,22].

With regard to the overshoot error caused by the switch, an adaptive hybrid intelligent control was adopted in Reference [23]. According to the changes of difference between system and external velocity, the damping coefficient was corrected in real time to keep the system errors caused by the ship’s maneuverability to a minimum. In Reference [24], a method of internal horizontal damping, based on the control of variable damping ratios was presented. It realized linear correction of the damping coefficients and effectively inhibited the overshoot error caused by the switch between the undamping status and damping status by changing the proportional control coefficient. In Reference [25], based on a detailed study and analysis of the overshoot phenomenon in the integrated calibration process of the platform inertial navigation system (PINS), the automatic compensation technique was adopted to reduce overshoot. In Reference [26], to address the problem of overshoot when the working status of the strapdown inertial navigation system (SINS) switched in the state of damping, an algorithm for overshoot error mitigation based on double mode was proposed.

In order to remedy for the defects of the fixed damping parameters, an adaptive damping network for the strapdown FOGC system was designed and implemented. By taking the horizontal velocity error as the objective function of the adaptive control, the optimal function between the damping coefficient and variation of the east component of velocity error of the damping network was established. According to the design principle of the two-order damping network, an adaptive damping network with timely variable damping coefficients was obtained using least square fitting. To address the problem of the overshoot error caused by status switching, an adaptive smooth switching technique is proposed in this paper. When the strapdown FOGC system switches from the undamping to damping status, the step size of the damping coefficient was adjusted to reduce the jump in the switching. The jump of the damping coefficient then transformed into a gradual change, thus allowing the overshoot caused by the change of damping network parameters to be effectively restrained. The effectiveness of the scheme was experimentally verified, and the dynamic performance of the strapdown FOGC system was obviously improved.

The outline of this paper is as follows: Section 1 is the introduction; the conventional external horizontal damping network and overshoot errors are reviewed briefly in Section 2; the proposed adaptive damping network designed for the strapdown FOGC for ships is provided in Section 3; Section 4 describes the technique used to restrain overshoot caused by the status switching of external damping; experimental results and discussions about the proposed method applied in the strapdown FOGC system are presented in Section 5, and Section 6 summarizes the conclusions.

## 2. The Conventional External Horizontal Damping Network and Overshoot Error

**Figure 1:**
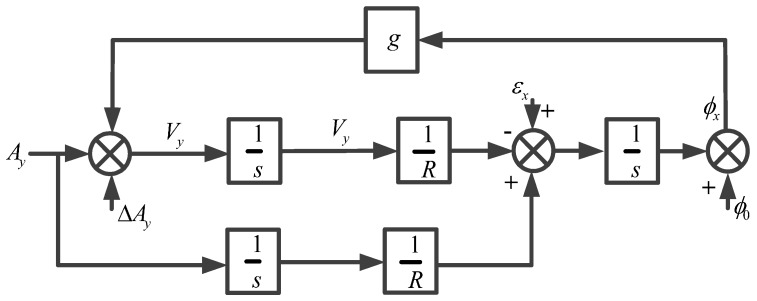
North-level correction circuit of the strapdown fiber optic gyrocompass （FOGC ）system [27,28].

If we take the north-level correction circuit of the strapdown FOGC system as an example, the horizontal loop model of the strapdown FOGC system is constructed [27,28] and is shown in Figure 1.

As shown in Figure 1, $A_{y}$ denotes the north component of acceleration; $\Delta A_{y}$ indicates the north component of velocity zero error; $V_{y}$ denotes the north component of velocity; $\varepsilon_{x}$ indicates the east component of gyro bias; $\phi_{0}$ denotes the initial misalignment angle; R is the Earth’s radius; g denotes gravitational acceleration; $\phi_{x}$ indicates the east component of misalignment angle; 1/s denotes integral operator.

We considered that gyro drift was the main error source of the strapdown FOGC system. In order to simplify the analysis, we assumed that only the influence of the east component of gyro drift was considered. The transfer function from gyro bias $\varepsilon_{x}$ to the horizontal error angle $\phi_{x}$ is defined as follows:

(1) $$\frac{\phi_{x}}{\varepsilon_{x}}=\frac{s}{s^{2}+g/R}=\frac{s}{s^{2}+\omega_{s}^{2}}$$

where $\omega_{s}=\sqrt{g/R}$ is the Schuler angular frequency.

**Figure 2:**
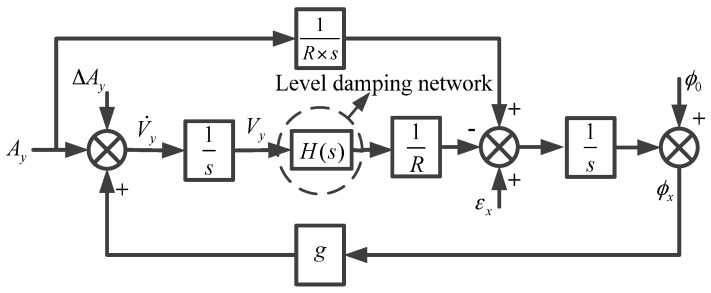
North component of level damping loop of strapdown FOGC system (conventional method).

With regard to the undamping strapdown FOGC system, it produces continuous oscillation or divergent errors under excitation of the constant gyro or random drift [27,28]. In order to restrain and attenuate the oscillation error of the strapdown FOGC (mainly referring to 84.4 min Schuler periodic oscillation), it is common to add the damping network H(s) into the forward channel of the correction circuit. The velocity information is the input into the damping network (shown in Figure 2). Changing the characteristic equation of the system to make the system have a negative real part eigenvalue, allows the strapdown FOGC system to stay in an asymptotically stable status [29,30].

After adding the damping network, the transfer function from gyro bias $\varepsilon_{x}$ to the horizontal error angle $\phi_{x}$ is

(2) $$\frac{\phi_{x}}{\varepsilon_{x}}=\frac{s}{s^{2}+\omega_{s}^{2}H(s)}.$$

As the damping network damages the condition of being without the interference Schuler tune, the transfer function from acceleration $A_{y}$ to the horizontal error angle $\phi_{x}$ is added into the strapdown FOGC system after adding the damping network, and is calculated as follows:

(3) $$\frac{\phi_{x}}{A_{y}}=\frac{1-H(s)}{s^{2}+\omega_{s}^{2}H(s)}.$$

In order to minimize the impact of maneuvering acceleration on the strapdown FOGC system, it is known from Equation (3) that damping network H(s) should be as close to 1 as possible; namely, the damping coefficient should be as small as possible. In contrast, in order to restrain and attenuate the oscillation error, it is known from Equation (2) that damping network H(s) makes the system have negative eigenvalues and provides a positive phase shift in the neighbor range of the Schuler angular frequency, $\omega_{s}$, and the larger the damping coefficient, the better. Considering the two above-mentioned conflicting aspects, the eclectic approach is commonly adopted in actual work, where around 0.5 is selected for the equivalent damping coefficient of the damping network. After repeated attempts, the parameters of network H(s) were adjusted to meet the requirements. The following two forms are generally adopted in the damping network [31,32].

(4) $$H(s)=\frac{(s+8.8\times 10^{-4}){\left(s+1.97\times 10^{-2}\right)}^{2}}{(s+4.41\times 10^{-3}){\left(s+8.8\times 10^{-3}\right)}^{2}},$$

(5) $$H(s)=\frac{\left(s+8.5\times 10^{-4})(s+9.412\times 10^{-2}\right)}{\left(s+8.0\times 10^{-3})(s+1.0\times 10^{-2}\right)}.$$

Equation (4) was selected as the damping network in this paper. When the strapdown FOGC system switches from the undamping status to the damping status, the circuit parameters mutate. According to the basic principle of automatic control, the output of the damping network under the action of step-input is

(6) $$Y(s)=\frac{(s+8.8\times 10^{-4}){\left(s+1.97\times 10^{-2}\right)}^{2}}{(s+4.41\times 10^{-3}){\left(s+8.8\times 10^{-3}\right)}^{2}}\frac{1}{s}.$$

The output of the damping network in time domain can be obtained after adopting an inverse Laplace transformation on Equation (6):

(7) $$y(t)=1-(0.0244t+9.7101)e^{-0.0088t}+9.7101e^{-0.0044t}.$$

**Figure 3:**
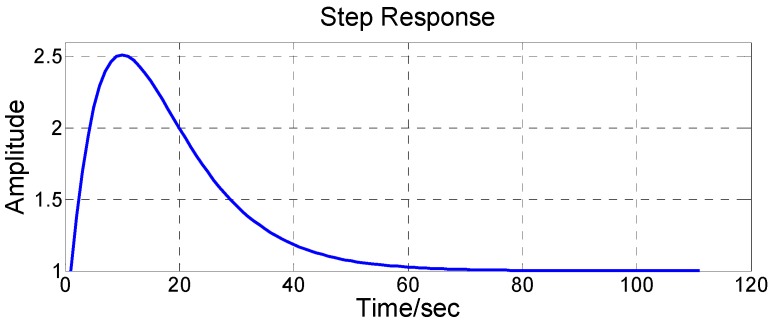
Response step of the damping network.

The response step of the damping network was analyzed from Equations (6) and (7), before the time domain response curve was obtained, as shown in Figure 3. It is known that the damping network in the time domain will overshoot under the action of input step.

**Figure 4:**
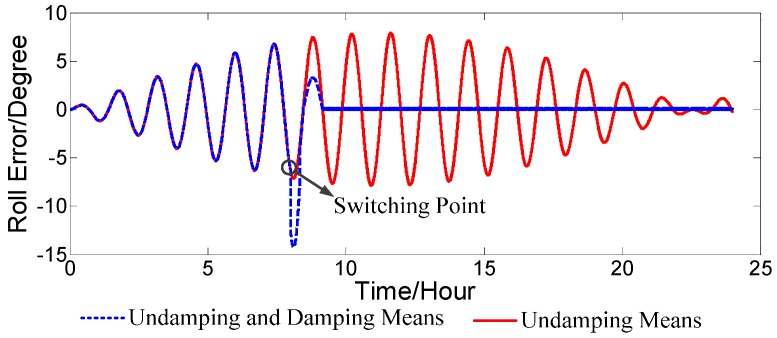
Switching overshoot without compensation.

After the overshoot output enters into the calculating loop, it causes an overshoot error of navigation parameters when the damping status of the strapdown FOGC system switches. In some cases, this kind of overshoot will be larger. The overshoot of the roll error, when the undamping status is switched to the damping status, is shown in Figure 4.

It can be seen in Figure 4 that the overshoot of the roll error was approximately two-fold when the statuses switched, and, in some cases, the overshoot may be even larger. A larger overshoot over a longer period will increase the error between the navigation parameter output from the strapdown FOGC system and the actual attitude of the carrier, which affects the reliability of the navigation parameters. This problem has not been resolved well in the conventional external horizontal damping network.

## 3. Design of the Adaptive Damping Network

### 3.1. The Control Scheme of the Adaptive Damping Network

**Figure 5:**
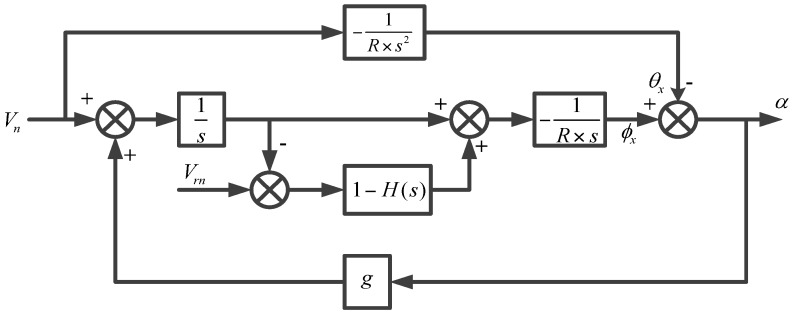
External horizontal damping block of single channel.

**Figure 6:**
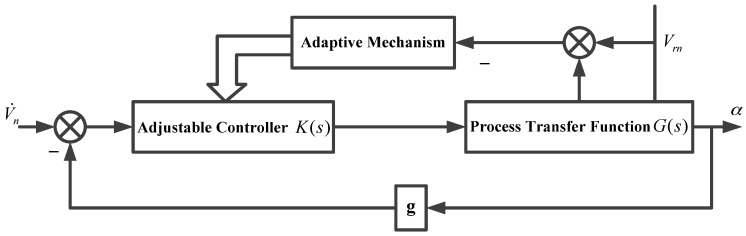
Adaptive control module of external velocity damping system.

The control scheme of the external horizontal damping block of a single channel was designed, as shown in Figure 5. The external velocity and system velocity were selected as the measured signals; the feed-forward adaptive control structure was adopted to control the damping network in real time; and the modified external horizontal damping block diagram is shown in Figure 6.

As shown in Figure 5, H(s) is the damping network; $V_{rn}$ is the north component of external velocity; ${\dot{V}}_{n}$ is the north component of acceleration; R is the Earth’s radius; g is the gravitational acceleration; α is the north component of the horizontal error angle; $\theta_{x}$ is the angle at which the geographic coordinate system rotates in the inertial space; $\phi_{x}$ is the angle at which the navigation coordinate system rotates in the inertial space.

In Figure 6, $K(s)=A/\left(S+\omega_{1}\right)+B/\left(S+\omega_{2}\right)$ is an adjustable parameter controller and $G(s)=1/RS^{2}$ is the process transfer function. The feed-forward adaptive control is also known as open loop adaptive control. With the help of measuring the process signal and through the adaptive mechanism (in accordance with the measured signal to change the state of the controller), the goal of changing the characteristics of the system was achieved [32,33]. The relationship between the measurement signal and the adjustable parameters of the controller can be established by the adaptive mechanism, which is the relationship between the system speed, the external speed signal, and the system equivalent damping coefficient.

### 3.2. The Relationship between the System Velocity, External Velocity, and the Damping Coefficient

We selected the system velocity error as the objective function of adaptive control:

(8) $$J=\int (\delta V_{e}^{2}+\delta V_{n}^{2})dt.$$

where $\delta V_{e}$, $\delta V_{n}$ are the east velocity error and the north velocity error in the system, respectively.

It is known from the principle of inertial navigation systems that the influence of the difference, δV, between system velocity and external velocity on the system error is closely related to the size of the equivalent damping coefficient. Supposing that the horizontal gyro drift of a certain type of strapdown FOGC system is $0.006^{o}/h$ and the azimuth gyro drift is $0.001^{o}/h$, the double loop optimization method was used for simulation in MATLAB. The specific methods are as follows: δV is fixed as a constant, the initial value of the damping coefficient is 0.01 and changes with a step of 0.01, and the optimal damping coefficient was obtained through the minimum objective function J. By changing the value of δV and repeating the above-mentioned steps, the optimal damping coefficient corresponding to all values of δV can be obtained. The approximate ideal corresponding relationship between $\delta V_{e}$ and damping coefficient ξ are shown in Table 1.

The relational expression can be obtained through the curve fitting data in Table 1 based on the least square fitting method.

(9) $$\xi =0.3076\delta V_{e}^{2}+1.1452\delta V_{e}+0.8101.$$

The interpolation models of external velocity and system velocity are as follows:

(10) $$\delta V_{e}=\delta V_{ce}+\delta V_{re}+k_{x}V_{e},$$

(11) $$\delta V_{n}=\delta V_{cn}+\delta V_{rn}+k_{y}V_{n}.$$

where $\delta V_{ce}$, $\delta V_{cn}$ are the constant errors of $\delta V_{e}$ and $\delta V_{n}$, respectively; $\delta V_{re}$, $\delta V_{rn}$ are the random errors of $\delta V_{e}$ and $\delta V_{n}$, respectively; $k_{x}$, $k_{y}$ are the maneuver coefficients of the ship, and their range is 0–1; and $V_{e}$, $V_{n}$ are the east component of velocity and the western velocity, respectively.

### 3.3. Adaptive Design of the Damping Network Parameters

In the event that the damping network of the system is [27,28]:

(12) $$H(s)=1+\frac{A}{s+\omega_{1}}+\frac{B}{s+\omega_{2}}$$

where A, B, $\omega_{1}$ and $\omega_{2}$ are parameters of the damping network.

Then, the characteristic equation of the system is

(13) $$s^{4}+(\omega_{1}+\omega_{2})s^{3}+(\omega_{1}\omega_{2}+\omega_{n}^{2})s^{2}+\omega_{n}^{2}(\omega_{1}+\omega_{2}+A+B)s+\omega_{n}^{2}(\omega_{1}\omega_{2}+A\omega_{2}+B\omega_{1})=0$$

where $\omega_{n}$ is the undamping natural oscillation frequency, $\omega_{n}=\sqrt{g/R}$.

Supposing that the dominant pole of the system is

(14) $$s_{1,2}=\sigma +j\omega_{d}$$

then the other two poles are

(15) $$s_{3}=n_{1}\sigma ,\;s_{4}=n_{2}\sigma \;n_{1},n_{2}\geq 5.$$

According to control theory,

(16) $$\omega_{d}=\omega_{n}\sqrt{1-\xi^{2}},\sigma =\omega_{n}\xi .$$

Then, the characteristic equation of the system is

(17) $$\begin{array}{l} s^{4}-(n_{1}+n_{2}+2)\omega_{n}\xi s^{3}+((n_{1}n_{2}+2(n_{1}+n_{2})+1)\omega_{n}^{2}\xi^{2}+\omega_{n}^{2}(1-\xi^{2}))s^{2}-\\ ((2n_{1}n_{2}+n_{1}+n_{2})\omega_{n}^{3}\xi^{3}+(n_{1}+n_{2})\omega_{n}^{3}(\xi -\xi^{3}))s++n_{1}n_{2}\omega_{n}^{4}\xi^{2}=0. \end{array}$$

When comparing Equation (16) with Equation (17), the following equations are obtained:

(18) $$\{\begin{array}{c} \omega_{1}=\frac{2b}{a+\sqrt{a^{2}-4b}}\\ \omega_{2}=\frac{b}{\omega_{1}}\\ A=\frac{\left(c-a)\omega_{1}-(d-b\right)}{\omega_{1}-\omega_{2}}\\ B=\frac{-A\omega_{2}}{\omega_{1}} \end{array}$$

where $a=-(n_{1}+n_{2}+2)\omega_{n}\xi$, $b=-\omega_{n}^{2}+(n_{1}n_{2}+2(n_{1}+n_{2})+1)\omega_{n}^{2}\xi^{2}+\omega_{n}^{2}(1-\xi^{2})$, $c=-((2n_{1}n_{2}+n_{1}+n_{2})\omega_{n}^{3}\xi^{3}+(n_{1}+n_{2})\omega_{n}^{3}(\xi -\xi^{3}))/\omega_{n}^{2}$, and $d=n_{1}n_{2}\omega_{n}^{4}\xi^{2}/\omega_{n}^{2}$.

Following the system stability criterion, we have:

(19) $$\left|n_{1}-n_{2}\right|\geq 9.$$

Taking Equation (15) into consideration, we can recommend $n_{1}=14,n_{2}=5$.

Substituting Equation (7) into Equation (18), we can obtain $\omega_{1}=f_{1}(\delta V_{e})$, $\omega_{2}=f_{2}(\delta V_{e})$, $A=f_{3}(\delta V_{e})$, $B=f_{4}(\delta V_{e})$. In this way, the controller parameters can be adjusted in real time according to the difference (δV) in the external velocity and the system velocity, but δV must be filtered to prevent large fluctuations in the damping parameters, resulting in an unstable system.

## 4. Suppression Technique of the Overshoot

From Schuler, Foucault, and the Earth circuit, the adjustment of the parameters is a slowly changing process. When the system switches working statuses, the introduction of interference will make the system produce the overshoot phenomenon. The navigation and positioning information provided by the system, which obviously has a large error in the time of system generated overshoot, will be analyzed in depth in this section.

### 4.1. Analysis of the Overshoot

**Figure 7:**
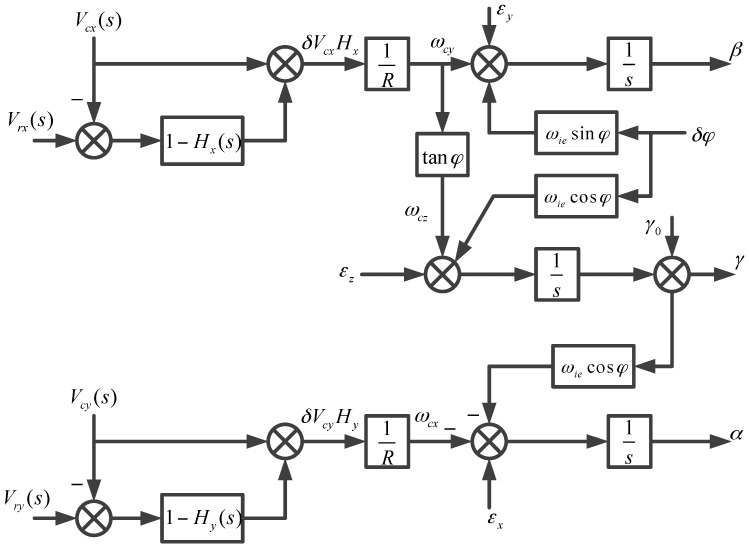
The simplified error principle diagram of strapdown FOGC.

When the strapdown FOGC system switches from the undamping status to the horizontal damping status, the damping network is introduced. The system no longer meets the Schuler adjusting condition, and the condition that the acceleration is without interference in the system is not fulfilled, so the equilibrium status is destroyed. The simplified error principle diagram of strapdown FOGC is shown in Figure 7.

As shown in Figure 7, $A_{x}$ is the acceleration along the east direction of the geographical coordinate system; $V_{cx}$ is the east component of velocity of the FOGC system; $V_{rx}$ is the east component of the external velocity; $H_{x}(s)$ is the east component of the horizontal damping network; $\omega_{ie}$ is the Earth rate.

It is supposed that the strapdown FOGC system switches from the undamping status to the external horizontal damping status at the time *t*_0_. When the system is using the undamping status, the corrected angular velocity is

(20) $$\omega_{cy}(t_{0}^{-})=\omega_{ie}\cos\varphi_{c}+\frac{V_{cx0}}{R}$$

where $V_{cx0}$ denotes the east component of velocity of the strapdown FOGC at the time of switching. The Schuler oscillation is the shortest period of oscillation in the undamping status; therefore, it can be considered that the east horizontal loop of the strapdown FOGC is stable at some point. Then, we obtain the following:

(21) $$\omega_{cy}(t_{0}^{-})+\varepsilon_{y}-\delta \varphi (t_{0}^{-})\omega_{ie}\sin\varphi_{c}=0$$

where $\varepsilon_{y}$ is the north component of the gyro bias. After the system switches to the external horizontal damping status, the corrected angular velocity of the gyro is as follows:

(22) $$\omega_{cy}(t_{0}^{+})=\omega_{ie}\cos\varphi_{c}+\frac{V_{cx0}}{R}+\frac{V_{rx0}-V_{cx0}}{R}[1-H_{x}(t)]=\omega_{cy}(t_{0}^{-})+\frac{V_{rx0}-V_{cx0}}{R}[1-H_{x}(t)]$$

where $V_{rx0}-V_{cx0}$ are the differences between the external velocity and the instantaneous value of the east velocity at the time of switching. When the system works using the undamping status, it is hard to ensure that $V_{rx0}-V_{cx0}=0$ because there is an error in the external velocity. Comparing Equation (20) to Equation (22), the corrected angular velocity mutates after the status switches. It is known that the equilibrium status of the whole east loop is broken when the angular rate mutates from Equation (21).

The same problem exists in the north loop and the azimuth loop. When the system switches from the undamping status to the damping status, the corrected angular rate mutates and the equilibrium status of the north and azimuth loop is broken, thus, overshoot occurs.

### 4.2. Overshoot Suppression of External Damping Status Switching

**Figure 8:**
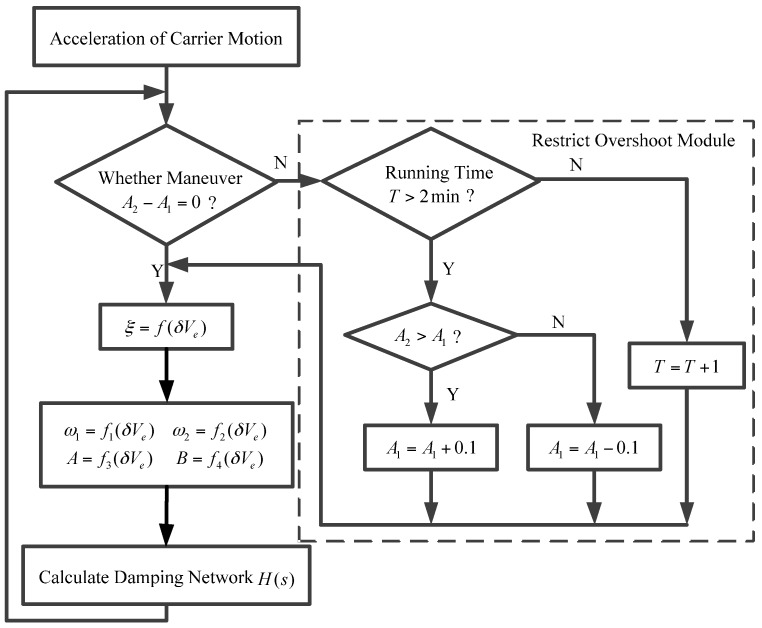
Calculation flow chart of an adaptive damping network.

The variation of the corrected angular velocity is proportional to the difference of the damping ratio before and after switching, and, at the same time, it is inversely proportional to the damping coefficient from the analysis results of external damping. An adaptive smooth switching technology, based on the above-mentioned analysis was put forward. When the strapdown FOGC system switches from the undamping status to the damping status, the changing step of damping coefficient ξ is adjusted to reduce the jumping change caused by the switch. The jumping change is transformed into the gradual change of the damping coefficient ξ, to try to make the system status switch smoothly. The design idea addressed: In order to reduce the overshoot due to the acceleration varies from $A_{1}$ to $A_{2}$, the damping coefficient corresponding to $A_{2}$ is adjusted by $A_{1}\pm 0.1$ every 2 min, until its value reaches the damping coefficient of $A_{2}$. A calculation flow chart of an adaptive damping network is shown in Figure 8.

**Figure 9:**
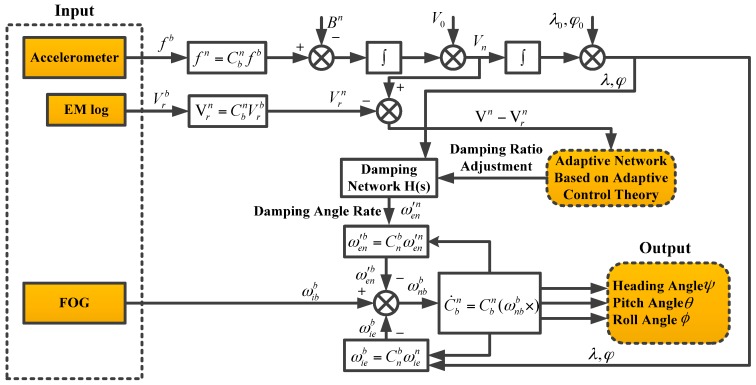
Block diagram of strapdown fiber optic gyrocompass.

The block diagram of the strapdown FOGC system is shown in Figure 9. Symbols *n* and *e* represent the navigation coordinate system and the Earth coordinate system, respectively. $V_{r}^{b}$ and $V_{r}^{n}$ denote the external velocity from the EM log in the body coordinate system and navigation coordinate system, respectively. The subscript 0 represents the initial value. $B^{n}$ and $f_{y}^{b}$ denote the disturbing acceleration and the output of accelerometer along the *y* axis, respectively. λ and φ indicate the longitude and latitude, respectively. $V^{n}$ represents the velocity of the strapdown FOGC system in the navigation coordinate system. $\omega_{AB}^{C}$ denotes the angular velocity of the *B* coordinate system relative to the *A* coordinate system, projected in the *C* coordinate system. ∫x and dx/dt represent the integral and differential of variable *x*, respectively.

As shown in Figure 9, the calculation flow chart of an adaptive network based on adaptive control theory is as shown in Figure 8. It is used to adjust the damping ratio for the damping network H(s) to make the system status switch smoothly.

## 5. Experimental Results and Discussions

In order to verify the effectiveness of the adaptive damping network, a great deal of simulation experiments and vehicle testing were carried out.

### 5.1. System Simulation Experiment

**Figure 10:**
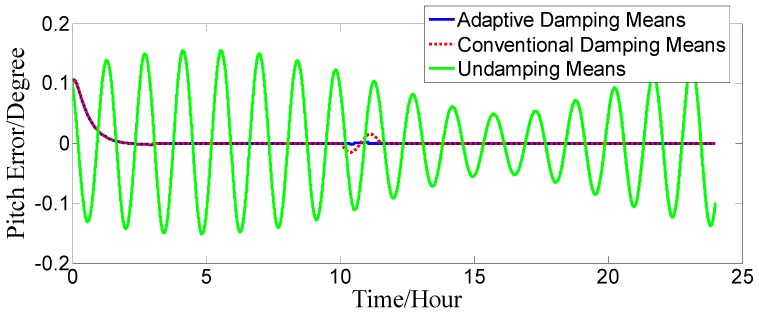
Pitch error.

**Figure 11:**
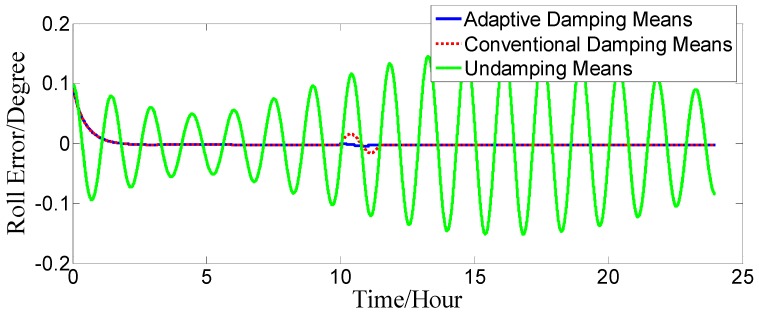
Roll error.

**Figure 12:**
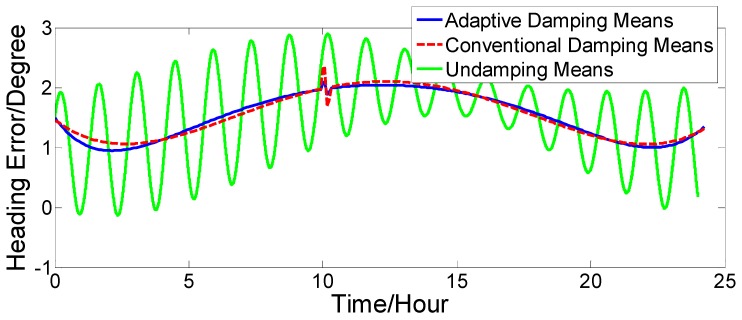
Heading error.

Supposing that the initial position was 32°03′52.93′′ N, 118°48′4.04′′ E, the gyro constant drift is $\varepsilon_{xc}=\varepsilon_{yc}=\varepsilon_{zc}=0.02^{o}/h$; the gyro random drift is $\varepsilon_{xr}=\varepsilon_{yr}=\varepsilon_{zr}=0.006^{o}/h$; the acceleration zero bias is $\Delta A_{x}=\Delta A_{y}=50\;\mu g$; the initial position error is $\delta \varphi_{0}=0.1^{\prime }$, $\delta \lambda_{0}=0.1^{\prime }$; the velocity constant error is $\delta V_{ce}=\delta V_{cn}=0.1\;\mathrm{kn}$; the velocity random error is $\delta V_{re}=\delta V_{rn}=0.2\;\mathrm{kn}$; and the initial error angle is $\phi_{x}=\phi_{y}=0.1^{o}$, $\phi_{z}=1.5^{o}$. The ship motion status is set as follows: when 0≤t<10 h, the ship sails straight at a constant velocity of V=2 kn and a heading of $H=45^{o}$. When 10 h≤t<10 h 2 min, the ship sails at variable acceleration and acceleration increases from 0 kn/min to 4 kn/min. When 10 h 2 min≤t<10 h 4 min, the acceleration decreases from 4 kn/min to 0 kn/min. At time t=10 h 4min, it switches to a constant velocity motion. The strapdown FOGC attitude error curves when the ship sails at a variable acceleration are shown in Figure 10, Figure 11 and Figure 12.

It is known from theoretical analyses that, when the maneuverability of the carrier is larger, the system error of the undamping status is at a minimum; however, as the system works in the critical state, when disturbed, the system error easily diverges, but this working mode is rarely used on ships with long working times. However, when the external velocity error has larger changes in the single working mode of the external horizontal damping, the attitude error, velocity error and position error have large fluctuations, resulting in the so-called “bulge” phenomenon, and is rarely used in actual systems.

The dotted red line is the error curve of the strapdown FOGC, adopting the conventional damping network. The system emerges overshoot phenomenon and the system error is larger. The solid blue line is the error curve of the strapdown FOGC adopting the adaptive damping network, and its damping coefficient can be adjusted in a timely manner with changes of acceleration relative to the conventional damping network. The solid green line is the error curve of the strapdown FOGC adopting the undamping network. It can be seen in Figure 10, Figure 11 and Figure 12 that, when adopting the adaptive damping network, the oscillation amplitude of error angles caused by the ship’s maneuvering is smaller, and the dynamic performance of the system is obviously improved. When acceleration is reduced, the system gradually turned from strong damping to weak damping, and the overshoot is also very small as the method where the damping coefficient is gradually changed is adopted.

### 5.2. Vehicle Experiment

In order to verify the feasibility and reliability of the adaptive damping network designed in this study, a vehicle experiment was carried out on a relatively flat route in the Kowloon Lake campus of Nanjing Southeast University . In the vehicle experiment, position and homing inertial navigation system (PHINS) developed by the French iXBlue company and FOSN (inertial measurement unit (IMU)) developed by Casic33s were fixed together on a panel board and placed in the interior of the experimental vehicle. The parameters of our FOG and accelerometer are shown in Table 2. The update frequency of IMU data was 200 Hz. The constant drift, scale factors, cross coupling coefficient, installation error angle, etc., were calculated and compensated using the exact calibrations in References [33,34], so that these errors could all be ignored in the calibration.

**Figure 13:**
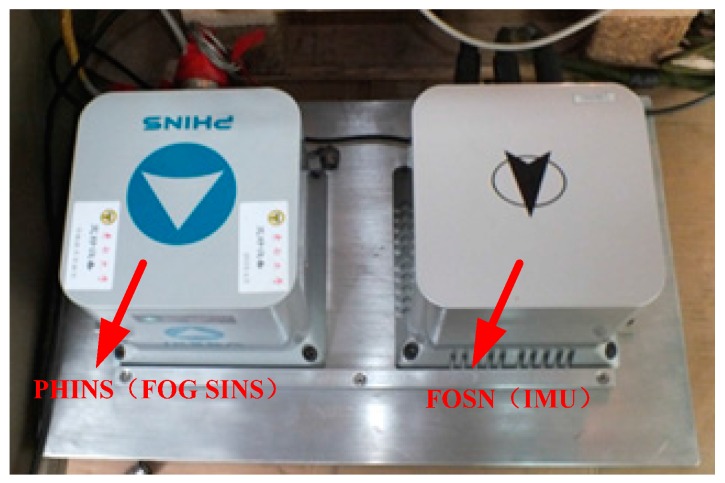
Equipment installation in the vehicle experiment.

**Figure 14:**
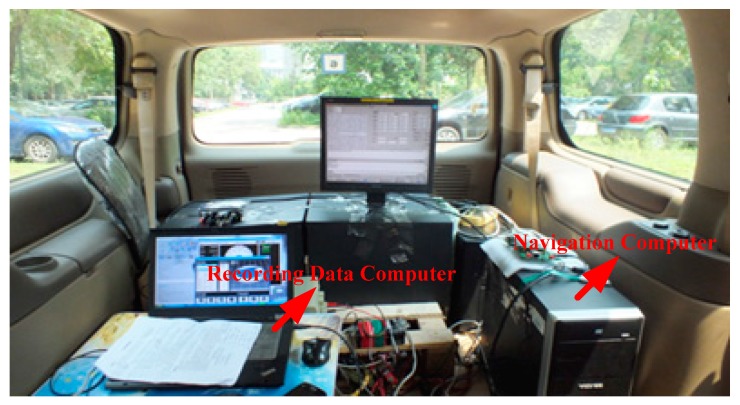
Interior environment in the vehicle experiment.

PHINS was set as the combination model with global position system (GPS), and the output of attitude information after PHINS and GPS, combined, was used as a reference index of vehicle navigation information. Equipment installation and the interior environment in the vehicle experiment are shown in Figure 13 and Figure 14.

**Figure 15:**
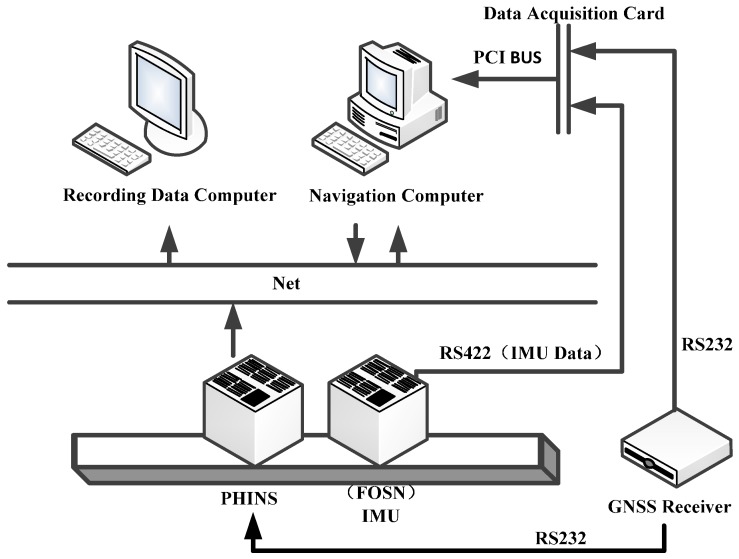
Schematic diagram of the vehicle experimental structure.

As shown in Figure 15, the experimental environment consisted of PHINS, IMU (FOSN), two computers, a global navigation satellite system (GNSS) receiver, data acquisition card, local area network, serial communication port, etc.

**Figure 16:**
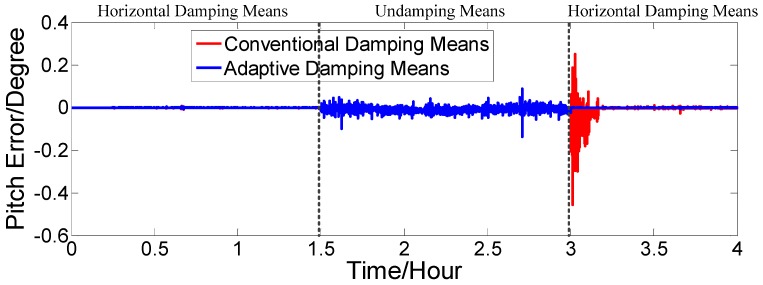
Pitch error.

**Figure 17:**
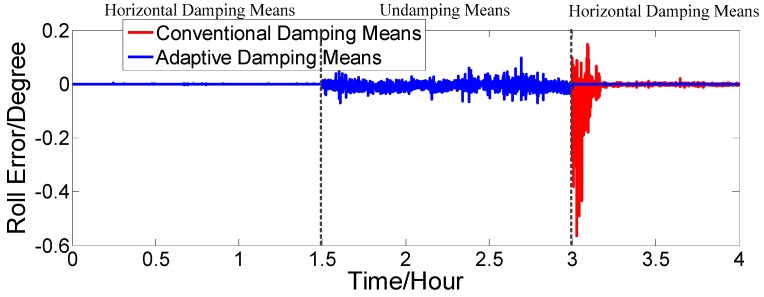
Roll error.

**Figure 18:**
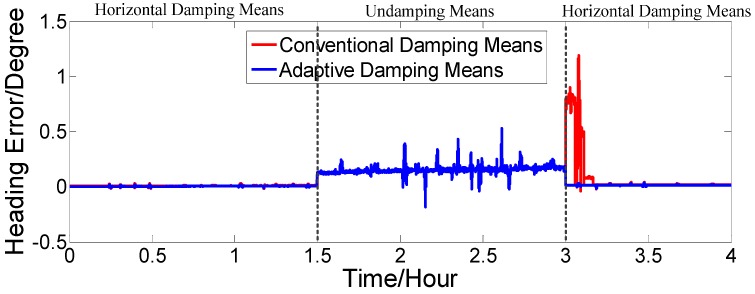
Heading error.

Figure 16, Figure 17 and Figure 18 show that our proposed adaptive damping method has a better performance than the conventional damping method. In our proposed method, the damping coefficient can be adjusted in a timely manner with changes of acceleration. Thus, oscillation amplitudes of attitude and heading angle errors decrease, while the overshoot (due to status switching) is very small. Furthermore, the attitude and heading angles of our proposed method are more accurate than the conventional one. Thus, the dynamic performance of the strapdown FOGC system is improved.

## 6. Conclusions

When the strapdown FOGC system for ships, adopting the external horizontal damping network, is in a dynamic case due to the influence of changing ocean currents and log disturbances, the oscillation amplitude of the system error is large, and switching from the undamping status to damping status will lead to a large overshoot error. Addressing the two major problems in the strapdown FOGC system, the adaptive control scheme of the external horizontal damping network was designed and realized. The difference in system velocity and log velocity were selected as the objective functions of adaptive control; the optimization function of variation of the damping coefficient with regard to velocity difference was established. According to the design principle of the two-order damping network, the adaptive damping network with variable damping coefficient was obtained by adopting the least square fitting. In order to restrain the overshoot error, the principle of overshoot phenomenon was analyzed, and the gradual changing technology of the damping coefficient was adopted to decrease the overshoot. Theoretical analyses and experimental results showed that the method can effectively reduce the oscillation amplitude of the system error and improve the dynamic performance of the strapdown FOGC system.

## Figures and Tables

**Table 1:** The corresponding relationship between $\delta V_{e}$ and damping coefficient ξ.

$\delta V_{e}/kn$	ξ
3.0	0.04
1.8	0.05
0.7	0.08
0.6	0.10
0.5	0.12
0.4	0.22
0.3	0.44
0.2	0.64
0.1	0.82
0.0	0.99

**Table 2:** Parameters of the fiber optic gyro (FOG)and accelerometer.

FOG		Accelerometer	
Constant errors	0.006°/h	Constant errors	50 µg
Random errors	0.006°/$\sqrt{h}$	Random errors	50 µg
